# Supplementary material for: The Novel AT2 Receptor Agonist β-Pro7-AngIII Exerts Cardiac and Renal Anti-Fibrotic and Anti-Inflammatory Effects in High Salt-Fed Mice
Source: Int J Mol Sci. 2022 Nov 14;23(22):14039. doi: 10.3390/ijms232214039 (PMC9696912; doi:10.3390/ijms232214039)
Supplement: Supplementary file 1 [file ijms-23-14039-s001.zip › ijms-2018355-supplementary.pdf]

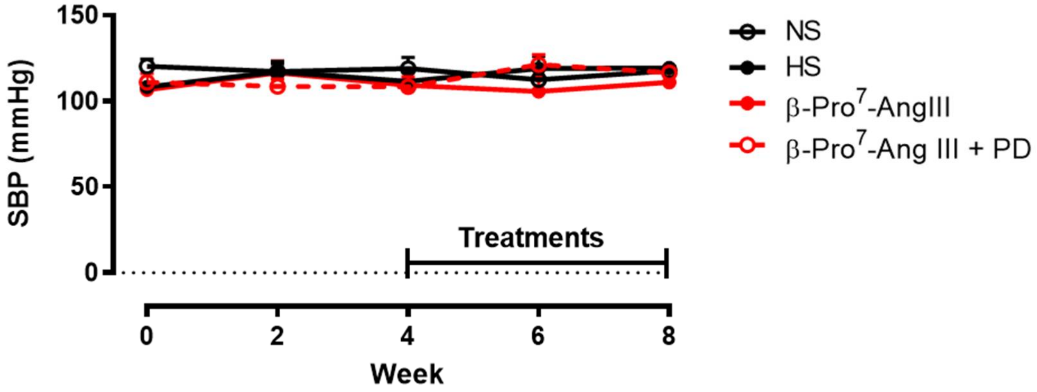

Figure S1. Effect of systolic blood pressure measured by tail cuff in male FVB/N mice fed a high salt (HS, 5% NaCl) diet for 8 weeks diet or mice fed on a high salt (HS, 5% NaCl) diet in the presence or absence of β-Pro^7-AngIII (0.1 mg/kg/day) +/- PD123319 (1mg/kg/day). All treatments were given for 4 weeks, between weeks 5-8 of HS diet. At the same time, a group of mice were fed on a normal salt (NS, 0.5% NaCl) diet for comparison. All data are expressed as mean ± s.e.m (n=6-8 per group).

Table S1. Primary and secondary antibodies for immunofluorescence staining.

Primary antibody	Cat #	Conc^n	Secondary antibody	Cat#	Conc^n
α-SMA rabbit pAb	Abcam (ab5694)	1:1000	Alexa Fluor® 594 Goat Anti-Rabbit IgG	Invitrogen (A-11037)	1:1000
TGF-β1 rabbit pAb	Abcam (ab92486)	1:500	Alexa Fluor® 594 Goat Anti-Rabbit IgG	Invitrogen (A-11037)	1:500
p-IκBα rabbit mAb	Cell Sig (#28595)	1:200	Alexa Fluor® 488 Goat Anti-Rabbit IgG	Invitrogen (A11008)	1:500
F4/80 macrophage rat mAb	Serotec (0505)	1:100	Alexa Fluor® 488 Goat Anti-Rat IgG	Invitrogen (A-11006)	1:500
MCP-1 rabbit pAb	Santa Cruz (sc-28879)	1:1000	Alexa Fluor® 488 Goat Anti-Rabbit IgG	Invitrogen (A11008)	1:500
